# Professional death avoidance and associated factors among intensive care unit nurses in China: a multicenter cross-sectional study

**DOI:** 10.3389/fmed.2026.1859295

**Published:** 2026-08-17

**Authors:** Ting Luo, Chaoya Hu, Ling Tang, Yuan Zhong, Yuzi Zhao, Huilan Wang, Yu Feng, Qian Yang

**Affiliations:** 1 School of Nursing, Chengdu Medical College, Chengdu, Sichuan, China; 2 Chengdu Second People’s Hospital, Chengdu, Sichuan, China; 3 Pengzhou Second People’s Hospital, Chengdu, Sichuan, China

**Keywords:** professional death avoidance, intensive care unit, nurses, emotional exhaustion, moral distress

## Abstract

**Background:**

Nurses in intensive care units (ICUs) frequently encounter patient death, ethical dilemmas, and emotionally demanding clinical situations. These experiences may be associated with emotional exhaustion, moral distress, and professional death avoidance. Professional death avoidance refers to a work-context-specific tendency to distance oneself from death-related professional situations, including communication, care tasks, emotional engagement, and reflection related to patient death. Understanding the current status of professional death avoidance and its associated factors is important for developing supportive strategies in critical care settings.

**Objectives:**

This study aimed to investigate the current status of professional death avoidance among ICU nurses and to examine its associations with emotional exhaustion, moral distress, and demographic and occupational characteristics.

**Methods:**

A multicenter cross-sectional study was conducted from September to October 2025 among 367 registered nurses from three tertiary hospitals in Sichuan Province, China. Participants completed validated questionnaires assessing professional death avoidance, emotional exhaustion, moral distress, and demographic and occupational characteristics. ICU work experience, job satisfaction, education level, monthly income, religious belief, ethics training, and family support were analyzed as potential associated factors. The Job Demands-Resources Model was used as a conceptual framework for variable selection and interpretation, rather than as an empirically tested theoretical model. Data were analyzed using descriptive statistics, univariate tests, Spearman rank correlation analysis, and exploratory stepwise multiple linear regression.

**Results:**

The mean professional death avoidance score was 38.26 ± 15.75. Univariate analysis showed significant differences in professional death avoidance scores according to ICU work experience, education level, monthly income, religious belief, ethics training, family support, and job satisfaction (*P* < 0.05). Professional death avoidance was positively correlated with emotional exhaustion (*r* = 0.447, *P* < 0.001) and moral distress (*r* = 0.526, *P* < 0.001). Exploratory multiple linear regression showed that ICU work experience, job satisfaction, emotional exhaustion, and moral distress were independently associated with professional death avoidance, collectively explaining 33.6% of the variance.

**Conclusion:**

ICU nurses in China reported a moderate level of professional death avoidance. Shorter ICU work experience, lower job satisfaction, higher emotional exhaustion, and higher moral distress were statistically associated with higher levels of professional death avoidance. These findings suggest that professional death avoidance may be related to both emotional depletion and ethical strain in ICU practice. Supportive workplace strategies addressing psychological well-being, moral distress, job satisfaction, and coping resources may help improve nurses’ engagement in death-related and end-of-life care, but longitudinal and intervention studies are needed to clarify causal relationships and evaluate intervention effectiveness.

## 1 Background

Death is an inevitable part of the life course, yet cultural attitudes toward death vary across societies (1, 2). Compared with relatively open discussions of death in many Western contexts, traditional Chinese culture often approaches death through implicit communication, emotional restraint, and avoidance. This cultural tendency creates a complex emotional environment in clinical settings, particularly in intensive care units (ICUs), where nurses frequently care for critically ill and dying patients (3).

ICU nurses are repeatedly exposed to patient suffering, critical illness, life-sustaining treatment decisions, and death, which may contribute to psychological outcomes such as emotional exhaustion and moral distress (4–6). Emotional exhaustion, the core dimension of burnout, refers to the depletion of emotional and physical resources caused by sustained work demands and is characterized by fatigue, reduced coping capacity, and psychological strain (7). Moral distress arises when nurses are unable to act in accordance with their professional and ethical judgment because of internal or external constraints, leading to conflict, guilt, and feelings of powerlessness (8, 9). These experiences are particularly salient in ICU settings, where nurses must provide technically demanding care while also facing emotionally and ethically challenging end-of-life situations.

In response to these stressors, ICU nurses may develop professional death avoidance. In this study, professional death avoidance refers to a work-context-specific behavioral tendency among ICU nurses to distance themselves from death-related professional situations, including communication about death, participation in death-related care tasks, emotional engagement with dying patients and their families, and reflection on death-related clinical experiences (10). This construct is not treated as synonymous with death anxiety, general avoidance coping, burnout-related disengagement, or end-of-life care avoidance. Death anxiety primarily denotes an affective response of fear, worry, or unease toward death (2). General avoidance coping reflects a broader coping style across stressful situations rather than a role-specific clinical behavior (11, 12). Burnout-related disengagement refers to a more generalized withdrawal from work and professional involvement (7). End-of-life care avoidance focuses more narrowly on reluctance to engage in palliative or terminal care activities. By contrast, professional death avoidance emphasizes nurses’ role-specific avoidance responses within ICU practice when death-related clinical, emotional, and ethical demands are encountered. Although such avoidance may provide temporary emotional protection, it may also reduce nurses’ engagement with patients and families and compromise the quality of end-of-life care (10).

Previous studies have examined ICU nurses’ perceptions of good death, death anxiety, coping with patient death, moral distress, burnout, and end-of-life care experiences (1–6, 10–12). These studies provide important evidence that repeated exposure to death and ethical dilemmas can affect nurses’ psychological well-being and professional responses. However, relatively few studies have treated professional death avoidance as a distinct professional behavioral construct. In particular, evidence remains limited regarding how professional death avoidance is associated with both emotional exhaustion and moral distress among ICU nurses in Chinese clinical settings. This gap is important because the Chinese cultural context, in which death is often discussed indirectly, may shape nurses’ professional responses to death-related care and may differ from findings reported in other cultural settings (2, 3, 11).

This study was guided by the Job Demands-Resources (JD-R) Model as a conceptual framework for interpretation rather than as an empirically tested theoretical model. The JD-R Model proposes that high job demands and insufficient job resources are associated with psychological strain and work-related behavioral outcomes (13, 14). In ICU nursing, repeated exposure to patient death, ethical dilemmas, high workload, and emotionally intense interactions may function as job demands, whereas ICU work experience, ethical support, job satisfaction, and family support may function as potential resources. Within this framework, professional death avoidance was conceptualized as a possible behavioral response associated with the imbalance between death-related job demands and available coping resources.

Therefore, this study aimed to: (1) assess the current status of professional death avoidance among ICU nurses in China; and (2) examine demographic, occupational, and psychological factors associated with professional death avoidance, including ICU work experience, emotional exhaustion, moral distress, and job satisfaction. By clarifying professional death avoidance as a distinct work-context-specific construct and examining its associated factors in Chinese ICU settings, this study may contribute to the international literature on ICU nurse well-being and provide evidence for developing culturally sensitive support strategies for nurses involved in end-of-life care.

## 2 Materials and methods

### 2.1 Study design and setting

A multicenter cross-sectional study was conducted from September to October 2025 in three public Grade-A tertiary hospitals in Sichuan Province, China. The participating hospitals were selected using a convenience-based approach, considering their tertiary hospital status, availability of ICU nursing staff, institutional willingness to participate, and feasibility of on-site data collection. All three hospitals provide critical care services and serve as regional tertiary medical centers. However, detailed institutional characteristics, such as ICU staffing models, bed capacity, end-of-life care policies, ethics consultation availability, and ICU subspecialty composition, were not systematically collected. The study protocol was approved by the Ethics Committee of Chengdu Medical College (Approval No. CMCIR2025NO.044) and was conducted in accordance with the Declaration of Helsinki.

### 2.2 Study participants

A total of 400 ICU nurses were invited to participate through department announcements and emails distributed by ICU head nurses. Recruitment was convenience-based and voluntary. Eligible nurses from ICU departments of the participating hospitals were invited to complete the questionnaire. The inclusion criteria were: (1) on-the-job registered ICU nurses; (2) ability to read and complete the questionnaire independently; and (3) voluntary provision of written informed consent. The exclusion criteria were: (1) temporary absence due to illness or study leave; (2) diagnosed mental or psychological disorders; and (3) internship status.

The participating nurses worked in ICU-related clinical settings within the three hospitals. However, ICU subtype, such as medical ICU, surgical ICU, emergency ICU, cardiac ICU, or general ICU, was not collected as a separate stratification variable in the questionnaire. Therefore, differences in professional death avoidance across ICU subtypes could not be examined in the present study.

After informed consent was obtained, paper questionnaires were distributed and completed on site. Of the 400 questionnaires distributed, 33 were excluded because of incomplete or inconsistent responses. A total of 367 valid questionnaires were included in the final analysis, yielding a valid response rate of 91.75%.

### 2.3 Sample size calculation

The sample size was calculated according to the rule of thumb for multivariable regression: N ≥ 50 + 8 m, where m represents the number of predictors (15). With 20 potential predictors, the minimum required sample size was 210. After allowing for a 15% invalid response rate, at least 243 participants were required. The final sample of 367 nurses exceeded this requirement and provided sufficient statistical power.

### 2.4 Data collection

Data were collected using self-administered questionnaires that included the following measures.

General Information Questionnaire. This questionnaire was developed by the research team based on a literature review and expert discussion. It included 18 demographic and occupational variables: age, gender, marital status, educational background, department, only-child status, place of residence, ICU work experience, professional title, job level, ICU specialist nurse certificate, employment type, monthly income, religious belief, participation in ethics training during the previous year, family support, and current job satisfaction.

Professional Death Avoidance Scale for ICU Nurses. This scale was developed by Zhang Zhengmin et al. (16) in 2022 to operationalize professional death avoidance as a role-specific tendency among ICU nurses to avoid death-related professional situations. It contains 15 items rated on a 5-point Likert scale from 1 (“very inconsistent”) to 5 (“very consistent”). Total scores range from 15 to 75, with higher scores indicating higher levels of professional death avoidance among ICU nurses. In the original development and validation study conducted among Chinese ICU nurses, the scale demonstrated good internal consistency, with a Cronbach’s α coefficient of 0.953 (16). To our knowledge, evidence regarding its validation beyond the original Chinese ICU nurse population remains limited. Therefore, the scale was considered appropriate for the present Chinese ICU nurse sample, but its broader psychometric generalizability should be interpreted cautiously.

Emotional Exhaustion Scale. Emotional exhaustion was measured using the Emotional Exhaustion Subscale of the Maslach Burnout Inventory developed by Maslach et al. (7). The scale has demonstrated good reliability and validity among Chinese nurses (17). It includes 9 items rated on a 7-point Likert scale from 1 (“completely inconsistent”) to 7 (“completely consistent”). Total scores range from 9 to 63, with higher scores indicating more severe emotional exhaustion. In the present study, the Cronbach’s α coefficient was 0.906.

Moral Distress Scale for Nurses. Moral distress was measured using the Moral Distress Scale developed by Corley et al. (18) and revised by Hamric et al. (19). The Chinese version translated and localized by Sun Xia et al. (20) was used in this study. The scale contains 22 items across four dimensions: individual responsibility (8 items), failure to uphold patients’ best interests (5 items), value conflict (6 items), and harm to patients’ interests (3 items). Each item includes two components: frequency and degree of distress, both rated on a 5-point Likert scale. The item score is calculated as the product of frequency and distress, and total scores range from 0 to 352. Higher scores indicate greater moral distress. In the present study, the Cronbach’s α coefficient was 0.879.

Researchers provided on-site explanations and assistance to ensure correct questionnaire completion. Participants were informed of the study objectives, voluntary participation, and confidentiality procedures. All data were double-entered and cross-checked by two independent researchers. Questionnaires with missing or logically inconsistent responses were excluded before analysis.

### 2.5 Statistical analysis

Data were analyzed using R version 4.4.0 (R Foundation for Statistical Computing, Vienna, Austria). Normality of continuous variables was assessed using the Shapiro-Wilk test. Normally distributed continuous variables were expressed as mean ± standard deviation (SD), whereas non-normally distributed variables were expressed as median and interquartile range. Categorical variables were expressed as frequency (n) and percentage (%).

Differences in professional death avoidance scores across categorical variables were examined using one-way analysis of variance for normally distributed data and Kruskal-Wallis tests for non-normally distributed data. Spearman rank correlation analysis was used to examine associations among professional death avoidance, emotional exhaustion, and moral distress.

The JD-R Model informed the selection and interpretation of variables, but the present cross-sectional design did not aim to empirically test the full JD-R model. Variables considered for multivariable regression analysis were selected based on both statistical significance in the univariate analyses and theoretical relevance according to the JD-R framework and previous literature. Specifically, variables that were statistically significant in the univariate analyses, together with theoretically important variables including ICU work experience, job satisfaction, emotional exhaustion, and moral distress, were considered as candidate variables.

Stepwise multiple linear regression was used as an exploratory approach to identify factors statistically associated with professional death avoidance. This approach was chosen because the study included multiple demographic, occupational, and psychological variables, and the aim was to explore the relative contribution of candidate variables while reducing the number of variables retained in the final model. We acknowledge that stepwise regression is data-driven and may increase the risk of model instability, overfitting, and biased coefficient estimates. Therefore, the regression results were interpreted cautiously as exploratory associations rather than confirmatory evidence.

Model residuals were assessed for normality, homoscedasticity, and independence. Variance inflation factor (VIF) values less than 2 indicated no multicollinearity. For the regression analysis, unstandardized regression coefficients (B), 95% confidence intervals (CIs), standard errors (SEs), standardized coefficients (β), t values, *P* values, and VIF values were reported. Statistical significance was defined as a two-tailed *P* value < 0.05.

## 3 Results

### 3.1 Participant characteristics and univariate analysis

A total of 367 ICU nurses were included in the final analysis. The sample comprised 61 males (16.6%) and 306 females (83.4%), with a mean age of 31.38 ± 5.30 years. Most participants held a bachelor’s degree (80.9%) and were contract employees (84.2%).

Regarding ICU work experience, 65 nurses (17.7%) had less than 1 year of experience, 132 (36.0%) had 1–5 years, 101 (27.5%) had 6–10 years, and 69 (18.8%) had more than 10 years of experience.

Univariate analysis showed that professional death avoidance scores differed significantly by ICU work experience, education level, monthly income, religious belief, ethics training, family support, and job satisfaction (*P* < 0.05). Nurses with shorter ICU work experience and lower job satisfaction tended to report higher levels of professional death avoidance. Detailed results are presented in Table 1.

**TABLE 1:** Univariate analysis of professional death avoidance by demographic and occupational characteristics among ICU nurses (*n* = 367).

Characteristic	*n* (%)	Professional death avoidance score, mean ± SD	Statistic	*P*
Demographic characteristics				
Gender			0.911	0.363
Male	61 (16.6)	39.93 ± 17.29		
Female	306 (83.4)	37.92 ± 15.43		
Age, years			1.265	0.286
18–25	39 (10.6)	38.59 ± 17.38		
26–35	254 (69.2)	38.56 ± 15.23		
36–45	65 (17.7)	35.82 ± 16.18		
> 45	9 (2.5)	45.89 ± 19.23		
Marital status			0.251	0.778
Married	227 (61.9)	38.25 ± 15.18		
Unmarried	133 (36.2)	38.48 ± 16.10		
Other	7 (1.9)	34.14 ± 18.96		
Education level			3.956	0.009
Technical secondary school	9 (2.5)	41.44 ± 19.71		
College	51 (13.9)	41.47 ± 14.30		
Bachelor’s degree	297 (80.9)	37.14 ± 15.71		
Master’s degree or above	10 (2.7)	52.00 ± 13.12		
Only child			0.023	0.982
Yes	139 (37.9)	38.28 ± 15.92		
No	228 (62.1)	38.24 ± 15.68		
Residence			0.524	0.601
Urban	221 (60.2)	38.61 ± 16.71		
Rural	146 (39.8)	37.73 ± 14.21		
Occupational characteristics				
ICU work experience			4.562	0.004
< 1 year	65 (17.7)	40.97 ± 15.95		
1–5 years	132 (36.0)	41.00 ± 16.86		
6–10 years	101 (27.5)	35.78 ± 14.95		
> 10 years	69 (18.8)	34.07 ± 13.08		
Professional title			1.166	0.323
Nurse	39 (10.6)	41.79 ± 17.60		
Senior nurse	164 (44.7)	38.62 ± 15.26		
Supervisor nurse	151 (41.1)	36.80 ± 15.44		
Director/deputy director nurse	13 (3.5)	39.92 ± 19.19		
Job level			1.639	0.164
N0	31 (8.4)	42.65 ± 16.81		
N1	73 (19.9)	39.44 ± 16.44		
N2	156 (42.5)	38.41 ± 15.36		
N3	95 (25.9)	35.32 ± 14.92		
N4	12 (3.3)	41.00 ± 18.41		
Employment type			2.367	0.071
Permanent staff	35 (9.5)	41.00 ± 15.11		
Contract employee	309 (84.2)	37.64 ± 15.61		
Labor dispatch	9 (2.5)	34.22 ± 18.49		
Other	14 (3.8)	47.57 ± 16.37		
Monthly income			4.220	0.006
<6000 yuan	147 (40.1)	41.18 ± 15.32		
6000–8000 yuan	151 (41.1)	36.93 ± 15.74		
8000–10,000 yuan	48 (13.1)	32.71 ± 15.13		
>10,000 yuan	21 (5.7)	40.00 ± 16.68		
Training, belief, support, and job satisfaction				
Ethics training			0.603	0.040
None	107 (29.2)	40.89 ± 15.79		
Received	260 (70.8)	37.17 ± 15.64		
Religious belief			2.963	0.003
Yes	27 (7.4)	29.70 ± 14.52		
No	340 (92.6)	38.94 ± 15.66		
Family support			6.102	<0.001
No support	61 (16.6)	41.34 ± 15.94		
Limited support	35 (9.5)	44.23 ± 18.40		
Moderate support	133 (36.2)	39.52 ± 14.54		
Full support	138 (37.6)	34.16 ± 15.23		
Job satisfaction			11.300	<0.001
Very dissatisfied	24 (6.5)	48.08 ± 15.73		
Slightly dissatisfied	29 (7.9)	45.93 ± 15.70		
Neutral	149 (40.6)	40.51 ± 14.45		
Fairly satisfied	126 (34.3)	35.10 ± 15.36		
Very satisfied	39 (10.6)	28.08 ± 14.25		

SD, standard deviation. Professional death avoidance scores are presented as mean ± SD. The statistic column reports the corresponding test statistic from the univariate comparison. *P* < 0.05 were considered statistically significant.

### 3.2 Descriptive statistics of key variables

The mean professional death avoidance score among ICU nurses was 38.26 ± 15.75, indicating a moderate level. The mean emotional exhaustion score was 30.53 ± 14.67, and the mean moral distress score was 82.53 ± 74.18.

### 3.3 Correlation analysis of professional death avoidance, emotional exhaustion, and moral distress

Spearman rank correlation analysis showed that professional death avoidance was positively correlated with emotional exhaustion (*r* = 0.447, *P* < 0.001) and moral distress (*r* = 0.526, *P* < 0.001). These findings indicate that higher levels of emotional exhaustion and moral distress were associated with greater professional death avoidance among ICU nurses.

### 3.4 Multivariate regression analysis

Exploratory stepwise multiple linear regression was conducted to examine factors statistically associated with professional death avoidance. Variables that were statistically significant in the univariate analysis, together with theoretically relevant variables including ICU work experience, job satisfaction, emotional exhaustion, and moral distress, were considered as candidate variables for the regression model.

The regression model showed that ICU work experience, job satisfaction, emotional exhaustion, and moral distress were independently associated with professional death avoidance. ICU work experience was negatively associated with professional death avoidance (*B* = −2.070, 95% CI: −3.557 to −0.583, β = −0.130, *P* = 0.006), indicating that nurses with longer ICU work experience tended to report lower levels of professional death avoidance. Job satisfaction was also negatively associated with professional death avoidance (*B* = −1.706, 95% CI: −3.295 to −0.117, β = −0.108, *P* = 0.035).

Emotional exhaustion was positively associated with professional death avoidance (*B* = 0.166, 95% CI: 0.044 to 0.288, β = 0.155, *P* = 0.007), and moral distress showed the strongest positive association with professional death avoidance in the model (*B* = 0.083, 95% CI: 0.061 to 0.105, β = 0.393, *P* < 0.001). These findings indicate that higher levels of emotional exhaustion and moral distress were statistically associated with higher levels of professional death avoidance among ICU nurses.

The overall model was statistically significant (*F* = 24.181, *P* < 0.001) and explained 33.6% of the variance in professional death avoidance (adjusted R^2^ = 0.336). No significant multicollinearity was detected, with all VIF values less than 2. Detailed regression results are presented in Table 2.

**TABLE 2:** Exploratory stepwise multiple linear regression of factors associated with professional death avoidance among ICU nurses.

Predictor	B	95% CI for B	SE	β	t	*P*	VIF
Constant	28.605	12.404 to 44.806	8.238	–	3.472	0.001	–
ICU work experience	−2.070	−3.557 to −0.583	0.756	−0.130	−2.740	0.006	1.247
Job satisfaction	−1.706	−3.295 to −0.117	0.808	−0.108	−2.112	0.035	1.439
Emotional exhaustion	0.166	0.044 to 0.288	0.062	0.155	2.690	0.007	1.827
Moral distress	0.083	0.061 to 0.105	0.011	0.393	7.624	< 0.001	1.467
Education level	0.874	−1.895 to 3.643	1.408	0.027	0.620	0.536	1.055
Monthly income	−0.398	−2.156 to 1.360	0.894	−0.022	−0.445	0.657	1.311
Religious belief	4.413	−0.710 to 9.536	2.605	0.073	1.694	0.091	1.031
Ethics training	−1.012	−3.978 to 1.954	1.508	−0.029	−0.671	0.503	1.047

B, unstandardized regression coefficient; CI, confidence interval; SE, standard error; β, standardized regression coefficient; VIF, variance inflation factor. The dependent variable was professional death avoidance. The model was statistically significant, *F* = 24.181, *P* < 0.001, adjusted R^2^ = 0.336.

## 4 Discussion

### 4.1 Current status of professional death avoidance among ICU nurses

This study found that ICU nurses in China reported a moderate level of professional death avoidance, with a mean score of 38.26 ± 15.75. Nurses with shorter ICU work experience, particularly those with less than 1 year or 1–5 years of experience, tended to report higher levels of professional death avoidance, whereas nurses with more than 10 years of ICU experience reported lower scores. These findings indicate that professional death avoidance differs according to ICU work experience and may be more prominent among less experienced nurses who have not yet developed sufficient coping strategies for death-related clinical situations.

Beyond describing the current status of professional death avoidance, the present study adds to previous research in several ways. First, whereas earlier studies have mainly focused on death anxiety, coping with patient death, burnout, moral distress, or end-of-life care attitudes among ICU nurses (1–6, 10–12), this study examined professional death avoidance as a distinct work-context-specific behavioral construct. Second, by simultaneously examining emotional exhaustion and moral distress, this study provides empirical evidence that professional death avoidance is associated with both emotional depletion and ethical strain in ICU nursing practice. Third, the findings offer culturally contextualized evidence from Chinese ICU settings, where indirect communication about death may shape nurses’ professional responses to death-related care. Therefore, the added value of this study lies not only in confirming associations that are theoretically consistent with the JD-R framework, but also in clarifying how death-related psychological and ethical burdens are reflected in nurses’ professional avoidance behaviors within a specific cultural and clinical context.

The moderate level observed in this study is consistent with previous research showing that ICU nurses may use avoidance-related responses when repeatedly exposed to patient death and high-intensity clinical demands (21, 22). In the Chinese context, cultural norms that discourage open discussion of death may further reinforce professional death avoidance. Nurses may feel constrained when discussing death with patients’ families or colleagues, making avoidance a more likely professional response in death-related care situations (23).

Education level and job satisfaction were also associated with professional death avoidance in the univariate analysis. Nurses with higher educational preparation may have more theoretical knowledge, ethical awareness, and communication skills for managing end-of-life situations (24). Similarly, nurses with higher job satisfaction may perceive greater organizational support and professional value, which can serve as protective resources when facing death-related stressors (25). However, because these variables did not all remain statistically significant in the multivariable model, these findings should be interpreted cautiously and require further confirmation in future studies.

Religious belief was statistically significant in the univariate analysis but was not independently associated with professional death avoidance in the multivariable regression model. This finding should be interpreted cautiously. One possible explanation is the small number of participants reporting religious belief in this sample, which may have limited the statistical power to detect an independent association after adjustment. In addition, the association observed in the univariate analysis may have been partly confounded by other occupational and psychological factors, such as ICU work experience, job satisfaction, emotional exhaustion, or moral distress. From a cultural perspective, the meaning and role of religiosity in death-related coping may differ across societies. In China, religious belief may not function in the same way as in contexts where formal religious practices are more closely integrated into end-of-life care and bereavement support. Moreover, this study measured religious belief only as a binary variable and did not assess religious affiliation, intensity of belief, spiritual coping, or death-related beliefs. Future studies should use more detailed measures of religiosity and spirituality to clarify their relationship with professional death avoidance.

From a JD-R perspective, the association between ICU work experience and professional death avoidance may reflect differences in nurses’ available coping resources when facing death-related job demands. Less experienced nurses may encounter substantial demands, including exposure to patient death, critical events, ethical challenges, and communication with patients’ families, while having fewer personal and professional resources to manage these situations. This demand-resource imbalance may be associated with greater emotional strain and a higher tendency toward avoidance-related professional behaviors. In contrast, nurses with longer ICU work experience may have accumulated more clinical knowledge, communication skills, emotional regulation strategies, and confidence in managing death-related care. These resources may help them remain more engaged in end-of-life care situations (10, 26). However, because the present study used a cross-sectional design, this interpretation should be understood as a JD-R-informed explanation rather than evidence of a causal pathway.

Overall, these findings highlight the multifactorial nature of professional death avoidance. It may be related to individual characteristics, occupational conditions, cultural context, and the psychological burden of high-stress ICU work. The moderate level identified in this study suggests the need for targeted support, particularly for less experienced nurses, to strengthen coping capacity and improve engagement in end-of-life care.

### 4.2 Emotional exhaustion and moral distress associated with professional death avoidance

Emotional exhaustion and moral distress were positively associated with professional death avoidance among ICU nurses. Nurses with higher emotional exhaustion scores tended to report higher levels of professional death avoidance, suggesting that emotional depletion may be related to reduced capacity to engage with patient death, end-of-life communication, and death-related professional tasks. This finding is consistent with previous research showing that burnout, particularly emotional exhaustion, is associated with reduced professional engagement and avoidance-related responses in ethically and emotionally challenging care situations (6, 27).

Moral distress showed the strongest positive association with professional death avoidance in the multivariable regression model. When nurses perceive that they are unable to act in accordance with their professional and ethical judgment, they may experience internal conflict, guilt, or powerlessness. In such situations, professional death avoidance may function as an avoidance-related response associated with the emotional and ethical burden of death-related care (28). This finding suggests that professional death avoidance should not be understood only as an individual coping style, but also as a professional behavioral response that may be linked to ethically constrained clinical environments.

These associations can be interpreted within the JD-R framework. In ICU nursing, repeated exposure to patient death, ethical dilemmas, heavy workloads, life-sustaining treatment decisions, and emotionally intense interactions with patients and families may represent important job demands. When psychological, ethical, and organizational resources are insufficient, nurses may be more likely to experience emotional exhaustion and moral distress. Professional death avoidance may therefore represent an avoidance-related behavioral response associated with emotional depletion and ethical strain under high-demand ICU conditions (14, 29). Nevertheless, the JD-R Model was used in this study as a conceptual framework for interpreting the observed associations rather than as an empirically tested theoretical model. Therefore, the present findings should not be interpreted as confirming a causal JD-R pathway.

The findings are consistent with studies conducted in high-acuity care environments internationally (30, 31). ICU nurses who frequently encounter patient mortality, withdrawal of life-sustaining treatment, and ethical dilemmas tend to report higher psychological burden and avoidance-related responses. In Chinese ICU settings, cultural norms surrounding indirect discussion of death may further intensify these associations by making it more difficult for nurses to express ethical concerns or discuss death-related issues openly.

These findings have practical implications for ICU nursing management. Supportive strategies should address both emotional exhaustion and moral distress by improving workload management, providing psychological support, and establishing structured debriefing after patient death or other critical events. Ethics consultation services, reflective practice sessions, and regular case discussions may also help nurses process difficult experiences and reduce avoidance-related professional responses. However, given the cross-sectional design of this study, these implications should be considered as directions for future intervention development rather than evidence of causal effects.

Although the regression model explained 33.6% of the variance in professional death avoidance, a substantial proportion of variance remained unexplained. This suggests that professional death avoidance is a complex phenomenon that may be associated with additional individual, clinical, organizational, and cultural factors not assessed in the present study. At the individual level, factors such as death anxiety, resilience, previous personal bereavement experiences, coping style, professional identity, and perceived competence in end-of-life communication may be relevant. At the clinical level, the frequency of patient death exposure, severity of patients’ conditions, frequency of withdrawal of life-sustaining treatment, and intensity of family-nurse communication may shape nurses’ responses to death-related care. At the organizational level, staffing levels, nurse-patient ratio, workload, team climate, psychological safety, ethics consultation availability, institutional end-of-life care policies, and access to structured debriefing or psychological support may also be associated with professional death avoidance. Cultural norms regarding death communication may further influence how nurses perceive and respond to death-related professional situations. Therefore, future studies should include broader individual, clinical, organizational, and cultural variables to provide a more comprehensive understanding of professional death avoidance among ICU nurses.

### 4.3 Implications for nursing practice

The findings of this study have several implications for ICU nursing management and professional development. Because this was a cross-sectional study, the following implications should be interpreted as potential directions for supportive practice and future intervention research rather than evidence of causal effects.

First, targeted support may be particularly important for less experienced ICU nurses. Structured mentorship programs, orientation training, supervised exposure to end-of-life care, and case-based learning may help novice nurses develop communication skills, emotional regulation strategies, and professional confidence when facing death-related clinical situations.

Second, strategies addressing emotional exhaustion may be useful for supporting ICU nurses’ psychological well-being. Workload optimization, flexible scheduling, peer support, resilience training, psychological counseling, and structured debriefing after patient death or other critical events may help nurses maintain emotional resources and remain professionally engaged in death-related care situations. However, the effectiveness of these strategies in reducing professional death avoidance should be tested in future longitudinal or intervention studies.

Third, moral distress should be considered an important issue in ICU nursing management. Establishing ethics consultation services, reflective practice forums, and regular interdisciplinary case discussions may provide nurses with opportunities to discuss ethically challenging situations and process difficult clinical experiences. Creating a safe and supportive environment for ethical communication may help address the emotional and moral burden associated with death-related care.

Fourth, improving job satisfaction and workplace resources may be relevant to professional death avoidance. Recognition programs, professional development opportunities, participatory decision-making, and visible organizational support may strengthen nurses’ sense of professional value and belonging. These workplace resources may be particularly important in high-demand ICU environments where nurses repeatedly encounter patient death and complex end-of-life decisions.

Finally, supportive strategies should be culturally sensitive. In the Chinese cultural context, death is often discussed indirectly, which may make death-related communication more difficult for nurses, patients, and families. Death education and communication training should therefore acknowledge cultural norms while encouraging compassionate, professional, and appropriate communication about death and end-of-life care.

### 4.4 Limitations

This study has several limitations. First, the cross-sectional design limits the ability to infer causal or temporal relationships among ICU work experience, job satisfaction, emotional exhaustion, moral distress, and professional death avoidance. Therefore, the observed associations should not be interpreted as evidence that these factors cause professional death avoidance. Longitudinal studies are needed to clarify temporal relationships and potential causal pathways.

Second, all data were collected using self-reported questionnaires at a single time point, which may have introduced reporting bias, recall bias, social desirability bias, and common method bias. Although anonymity, voluntary participation, and confidentiality procedures were used to reduce response bias, the use of the same data source, the same measurement method, and the same collection time may have inflated the observed associations among professional death avoidance, emotional exhaustion, moral distress, and other self-reported variables. Therefore, the associations observed in this study should be interpreted cautiously. Future studies should consider using multi-source data, longitudinal designs, repeated measurements, or objective organizational indicators to reduce common method bias and strengthen the robustness of the findings.

Third, although a multicenter design was used, participants were recruited from only three public Grade-A tertiary hospitals in Sichuan Province, China. The hospitals were selected using a convenience-based approach according to institutional collaboration, feasibility of data collection, and willingness to participate. Therefore, selection bias may have been introduced at both the hospital and participant levels. In addition, institutional characteristics, such as ICU staffing patterns, workload, bed capacity, management culture, end-of-life care policies, ethics consultation availability, and psychological support resources, were not systematically assessed. These factors may have been associated with nurses’ experiences of death-related care, emotional burden, moral distress, and professional death avoidance. Furthermore, ICU subtype, such as medical ICU, surgical ICU, emergency ICU, cardiac ICU, or general ICU, was not collected as a separate stratification variable, so potential differences across ICU subtypes could not be examined. These issues limit the generalizability of the findings to other regions, hospital levels, institutional contexts, ICU subtypes, and non-ICU settings.

Fourth, the regression model explained 33.6% of the variance in professional death avoidance, indicating that additional factors were not captured in this study. Professional death avoidance may be associated with a broader range of individual, clinical, organizational, and cultural factors. Potentially relevant variables include death anxiety, resilience, coping style, previous bereavement experience, professional identity, perceived competence in end-of-life communication, nurse-patient ratio, frequency of patient death exposure, ICU subtype, withdrawal of life-sustaining treatment experience, end-of-life care policies, availability of ethics consultation, team climate, psychological safety, communication training, and institutional support mechanisms. Future studies should include these variables to provide a more comprehensive understanding of professional death avoidance.

Fifth, although validated instruments were used in this study, the psychometric evidence for the Professional Death Avoidance Scale remains relatively limited. This scale was originally developed and validated among Chinese ICU nurses, and evidence regarding its validation in other cultural contexts, healthcare systems, or non-ICU nurse populations is still insufficient. Therefore, the findings related to professional death avoidance should be interpreted in light of this measurement context. Future studies should further examine the scale’s psychometric properties, including construct validity, criterion validity, measurement invariance, and cross-cultural applicability in broader nursing populations. In addition, religious belief was measured only as a binary variable in this study. More detailed aspects of religiosity and spirituality, such as religious affiliation, strength of belief, spiritual coping, and attitudes toward death, were not assessed. This may have limited the interpretation of the relationship between religiosity and professional death avoidance.

Sixth, stepwise multiple linear regression was used as an exploratory modelling approach in this study. Although this method can help identify statistically associated factors among multiple candidate variables, it is data-driven and may be vulnerable to model instability, overfitting, and biased coefficient estimates. Therefore, the regression findings should be interpreted cautiously and should be confirmed in future studies using theory-driven modelling approaches, hierarchical regression, structural equation modelling, or longitudinal designs.

Finally, cultural attitudes toward death in China may have influenced participants’ responses and the manifestation of professional death avoidance. Because death is often discussed indirectly in Chinese cultural contexts, nurses’ avoidance-related professional behaviors may differ from those observed in settings with different cultural norms. Future cross-cultural studies are needed to examine whether the findings can be generalized to other sociocultural contexts.

## 5 Conclusion

This multicenter cross-sectional study found that ICU nurses in China reported a moderate level of professional death avoidance. ICU work experience, job satisfaction, emotional exhaustion, and moral distress were statistically associated with professional death avoidance. Nurses with shorter ICU work experience, lower job satisfaction, higher emotional exhaustion, and higher moral distress tended to report higher levels of professional death avoidance.

By distinguishing professional death avoidance from broader concepts such as death anxiety, general avoidance coping, burnout-related disengagement, and end-of-life care avoidance, this study provides culturally contextualized evidence on death-related professional behaviors among ICU nurses. The findings suggest that professional death avoidance may be understood as a work-context-specific behavioral response associated with death-related clinical demands, emotional depletion, ethical strain, and available coping resources in ICU practice. From a JD-R-informed perspective, these results may enrich international understanding of how ICU nurses respond professionally to patient death, particularly in cultural contexts where direct discussion of death is often limited.

The findings also highlight the need for supportive workplace strategies that address emotional exhaustion, moral distress, job satisfaction, and professional coping capacity among ICU nurses. However, because of the cross-sectional design, these strategies should be regarded as potential directions for future intervention development rather than evidence of causal effects. Future studies should use longitudinal or intervention designs and include broader, more diverse samples to clarify temporal relationships, examine additional individual, clinical, organizational, and cultural factors, and evaluate strategies for reducing professional death avoidance and improving nurses’ engagement in death-related and end-of-life care.

## Data Availability

The raw data supporting the conclusions of this article will be made available by the authors, without undue reservation.
